# Does sports therapy affect momentary affective states? Feasibility of intensive longitudinal case studies in forensic psychiatry

**DOI:** 10.3389/fpsyt.2023.1111602

**Published:** 2023-05-12

**Authors:** Vanessa Reimer, Martina K. Kanning

**Affiliations:** Department of Sport Science, Social and Health, Sciences University of Konstanz, Konstanz, Germany

**Keywords:** intensive longitudinal case studies, sports therapy, forensic psychiatry, affective states, energetic arousal, valence, calmness

## Abstract

Physical exercise interventions improve quality of life in people with mental disorders and improve abstinence and cravings in substance use disorders patients in both the short term and long term. In people with mental illness, physical exercise interventions significantly reduce psychiatric symptoms of schizophrenia and symptoms of anxiety. For forensic psychiatry, there is little empirical evidence supporting mental health-enhancing effects of physical exercise interventions. Interventional studies in forensic psychiatry deal mainly with three problems: heterogeneity of the individuals, a small sample size, and a low compliance rate. Intensive longitudinal case studies could be a suitable approach to address these methodological challenges in forensic psychiatry. This study uses an intensive longitudinal design to determine whether forensic psychiatric patients are content to complete several data assessments per day over the course of several weeks. The feasibility of this approach is operationalized by the compliance rate. Additionally, single-case studies examine the effects of sports therapy (ST) on momentary affective states (energetic arousal, valence, and calmness). The results of these case studies reveal one aspect of feasibility and offer insights into the effects of forensic psychiatric ST on the affective states among patients with different conditions. The patients’ momentary affective states were recorded before (PRE), after (POST) and 1 h after (FoUp1h) ST by questionnaires. Ten individuals (M_age_ = 31.7, SD = 11.94; 60% male) participated in the study. A total of 130 questionnaires were completed. To perform the single-case studies, data of three patients were considered. Repeated-measures ANOVA was performed for the individual affective states to test for main effects of ST. Due to the results, ST has no significant effect on none of the three affect dimensions. However, effect sizes varied between small to medium (energetic arousal: η^2^ = 0.01, η^2^ = 0.07, η^2^ = 0.06; valence: η^2^ = 0.07; calmness: η^2^ = 0.02) in the three patients. Intensive longitudinal case studies are a possible approach to address heterogeneity and the low sample size. The low compliance rate in this study reveals that the study design needs to be optimized for future studies.

## Introduction

There is a considerable amount of evidence showing that physical exercise interventions improve quality of life in people with mental disorders and improve abstinence and cravings in substance use disorder patients in both the short term and long term (1). In people with mental illness, physical exercise interventions significantly reduce psychiatric symptoms of schizophrenia (2) and symptoms of anxiety (3). A systematic review and meta-analysis investigating physical activity interventions in people with mental illness showed small to large short-term effects of physical activity on symptoms of mental illness (4). However, for forensic psychiatry, there is little empirical evidence supporting the effects of physical exercise interventions. Conducting research studies to investigate the effects of sports therapy (ST) on forensic psychiatric patients is challenging due to various reasons (5). Typical barriers include a low sample size in ST sessions, as patients from different wards have to train in separate groups and cannot be put into one group (5). Additional challenges include low compliance rates and high heterogeneity within psychiatric diagnoses (5). Furthermore, there are sociopsychological deficits associated with the crime that are specific to forensic psychiatry (6). Forensic patients suffer from poor physical conditions and a high variability and fluctuation in momentary affective states, which could be an explanation for the low sample size and the low compliance rate (5). In forensic patients, there are only a few patients who (can) participate in a study due to symptomatology and heterogeneity, baseline restrictions, nonparticipation, etc. (5), thereby causing variations and fluctuations in behavior and well-being (5). On average, a ST group in forensic psychiatry consists of a small number of participants (5–10) that fluctuates in each unit (5). Regarding intervention studies, the average compliance rate in forensic psychiatric studies is 53% (7), which is lower than the rate (63%) in people with serious psychiatric disabilities (8). The high heterogeneity in psychiatric diagnoses is due to a combination of several factors causing a mental illness such as individual characteristics and experiences, social influences, when certain events took place, or how the person developed (9). Challenges such as motivation (10) and impulsivity (10, 11) are additional factors that contribute to a low compliance rate and generally a low number of participants (7). These hurdles initially make it difficult to conduct studies that can then provide information on forensic patients’ responses to specific ST interventions that are aimed at improving well-being, quality of life and psychological functioning.

To address these challenges in forensic psychiatric research, using single-case studies could be used as a methodological solution. Single-case studies offer a useful method for evaluating data from patients who experience the heterogeneity of psychiatric disorders (12, 13), as individual variability as well as valuable information about the individual treatment progress can be lost in a between-group design (14). Intensive longitudinal case studies offer the ability to examine participants over a longer period of time (e.g., several weeks) and several times a day in their natural context (15). Therefore, a low sample size is accounted for in such designs. These types of studies require a higher amount of effort for researchers and participants, and it is unclear whether or to what extent this is tolerated by forensic patients due to motivational problems and impulsivity (5–7) and if the low compliance rate can be addressed. Data collection at multiple measurement timepoints is used to generate a sufficient amount of data per patient. The terms “single case study” and “N-of-1” study are used synonymously (12, 16, 17). Throughout this article, the term single-case study is used. A clear distinction should be drawn to a case report (17). A case report is purely descriptive, whereas a single-case study demonstrates rigorous methods for the study design and data analysis using visual and statistical methods whenever possible (17). A single-case study design is a common study design in health care (17–19), e.g., in psychiatric disorders, such as schizophrenia (13, 20, 21). This design addresses intraindividual differences (16, 17, 22) and contributes to individualized medicine (12, 23). The authors of a study in forensic psychiatry show, within a randomized controlled trial, that the addition of a single-case experimental design enables a more intense investigation of specific patients (24). They report the advantage of the single-case study designs in the form of closer monitoring of each patient and receiving valuable information about each patient (25) that is more in-depth (24). These findings suggest that single-case studies in forensic psychiatry could be used to address heterogeneity.

In the field of forensic psychiatry, case study designs have been implemented in the form of neurofeedback training (25) or music therapy (20), but none have specifically examined the effect of ST. This study used an intensive longitudinal design to determine whether forensic psychiatric patients are content to complete several data assessments per day over the course of several weeks. The feasibility of this approach was operationalized by the compliance rate. Additionally, single-case studies were performed to examine the effects of ST on momentary affective states (energetic arousal, valence, calmness). To the best of our knowledge, this is the first study in forensic psychiatry to evaluate the feasibility of an intensive longitudinal case study design. Furthermore, the results of these case studies reflect one aspect of feasibility and offer insights into the effects of forensic psychiatric ST on the affective states among patients with different conditions.

## Methods

### Sample and recruitment

Data were collected in 2021 at the Center for Psychiatry Reichenau. Approximately 100 patients are accommodated there and distributed across four forensic wards. People are referred to this center if their offense was specifically related to the use of intoxicating substances or mental illness (6). The total length of detention varies across individuals but ranges from a minimum of 2 years to multiple years (6). Participation in the study was voluntary and patients committed to submitting regular study data on days with ST (twice a week). The ST intervention represented the regularly occurring therapy and was firmly integrated at the Center for Psychiatry Reichenau. The repeated recording of the momentary affective states did not represent a psychological intervention. Prior to study inclusion, each subject signed an informed consent form. In total, 10 individuals participated in the study. The patients’ symptomatology included either paranoid schizophrenia, schizoaffective disorder (manic or mixed type), bipolar affective disorder (current episode manic without psychotic features), or mixed and other personality disorders or hyperkinetic conduct (Table 1). Participants received an organized bike tour as an incentive for participating in the study.

**Table 1:** Patient characteristics.

Patient	Sex	Diagnosis ICD-10	Data points total	Data points PRE	Data points POST	Data points FoUp1h
1^*^	M	F20.0 paranoid schizophrenia	27	10	10	7
2^*^	M	F20.0 paranoid schizophrenia	29	10	10	9
3	F	F61 mixed and other personality disorders	5	2	2	1
4	M	F20.0 paranoid schizophrenia	11	4	4	3
5	M	F25.0 schizoaffective disorder, manic type	12	5	5	2
6	F	F20.0 paranoid schizophrenia	9	3	3	3
7	M	F90.1 hyperkinetic conduct disorder	11	5	5	1
8	M	F20.0 paranoid schizophrenia	0	0	0	0
9	F	F25.2 schizoaffective disorder, mixed type	7	3	3	1
10^*^	F	F31.1 bipolar affective disorder, current episode manic without psychotic features	19	8	8	3

* Patients included for single-case analyses.

### Procedure

Questionnaires to assess demographic details were completed before the first session of ST. Over a period of four weeks, affective state questionnaires were completed before ST (PRE), after ST (POST) and 1 h after ST (FoUp1h). For the FoUp1h assessment timepoint, the sport therapist searched and contacted every patient in the facility.

### Measurements

Demographics. Age and gender were self-reported and assessed with a paper and pencil questionnaire.

Momentary affective states. Self-report was used to assess patients’ momentary affective states. For this purpose, the short scale by Wilhelm and Schoebi (26) was used (see Appendix 1). This short scale is appropriate to minimize the effort for the patients. This instrument is validated and suitable for ambulatory assessment studies. The within-person reliability (energetic arousal: 0.77, valence: 0.70, calmness: 0.77) as well as the between-person reliability (energetic arousal: 0.90, valence: 0.92, calmness: 0.90) was acceptable (26). Three bipolar affect dimensions (energetic arousal, valence, calmness) were measured by six items that assess the intensity of each affect dimension: energetic arousal (tired vs. awake, without energy vs. full of energy), valence (unwell vs. well, discontent vs. content), and calmness (relaxed vs. tense, calm vs. agitated). Responses were given on a 6-point Likert response scale.

### Intervention

All 60-min ST sessions followed exactly the same structure and the units were comparable in procedure, duration, and intensity. Patients in this study participated in two different ST groups. The main intended effects of ST are to increase physical fitness, promote social skills in group sports, and contribute to a solid daily structure (6). First, there was a warm-up phase that focused on circulation activation and technique practice. In the main phase, patients performed different group sport activities together (e.g., volleyball, soccer, tennis, or table tennis). The intensity of the activity was structured in such a way that it was perceived as hard by the individuals (27). Furthermore, ST included technical elements (throwing and passing techniques, running routes, team formations, etc.). The ST session ended with a collective cool-down, which was followed by a reflection phase in which the group discussed what went well during the session and what could be improved. Data collection took place in June and July 2021 at the Center for Psychiatry Reichenau.

### Data analysis

Intensive longitudinal single-case analyses were conducted to estimate whether forensic patients were content to complete several data assessments per day over the course of several weeks, with the feasibility being operationalized by the compliance rate (percentage of completed questionnaires). The longitudinal single-case analyses were also performed to address the methodological challenge of heterogeneity. In addition, differences between the three measurement timepoints (PRE, POST, FoUp1h) for each affect dimension (energetic arousal, valence, calmness) were examined. To analyze within-subject changes and to describe in more detail how ST affected momentary affective states, a one-way repeated-measures ANOVA was performed for the individual affective states. The ANOVA estimated if there were significant differences between the three measurement timepoints separately for each affect dimension. The weekly study sessions of ST as well as the factor time acted as independent variables, and the momentary affective states served as dependent variables.

Data were analyzed in RStudio with the corresponding describeBy() function from the psych package (28) (alpha level *p* < 0.05). For ANOVA, the aov() function of the afex package (29) was used. The effect sizes were calculated using the eta_squared() function from the effectsize package (30).

The requirements for ANOVA are the normal distribution of the data and variance homogeneity (31), which, as expected, was partially present in the dataset of this study. Nevertheless, repeated-measures ANOVA was conducted to give a certain trend recommendation.

## Results

### Descriptive results

A total of 130 questionnaires were completed (PRE: 50, POST: 50, FoUp1h: 30) by the 10 patients (*M*_age_ = 31.7, *SD* = 11.94; 60% male). Table 1 provides an overview of the data points provided by each patient (Table 1). The scores on the three affect dimensions were as follows (between-person distribution): energetic arousal, *M* = 4.6 (*SD* = 1.1; min = 1, max = 6); valence, *M* = 4.6 (*SD* = 1.47; min = 1, max = 6); and calmness, *M* = 4.9 (*SD* = 1.14; min = 1, max = 6).

### Single-case analyses

Patients 3 and 4 dropped out of the study after 10 and 14 days, respectively. Patient 8 did not participate in a single intervention measurement timepoint and consequently could not provide any conclusions about the effectiveness of ST. Due to the amount of missing data (≥40%), patients 5, 6, 7, and 9 were also excluded from the descriptive presentation of the results, as their incomplete data could not provide any conclusions about the effectiveness of ST. Therefore, a statistical presentation of the results of patient 1 (PRE: *N* = 10, POST: *N* = 10, FoUp1h: *N* = 7), patient 2 (PRE: *N* = 10, POST: *N* = 10, FoUp1h: *N* = 9), and patient 10 (PRE: *N* = 8, POST: *N* = 8, FoUp1h: *N* = 3) was included for analyses (compliance rate: >63%). The effect size is an important finding to report, as a *p* value only indicates the presence of an effect, but does not indicate the size of the effect (32). Effect sizes, unlike significance tests, are not dependent on sample size, but a significance test does (32). When describing the effect sizes, the following values are considered: η^2^ = 0.01 (small effect), η^2^ = 0.06 (medium effect), and η^2^ = 0.14 (large effect) (33).

### Patient 1

**Figure 1:**
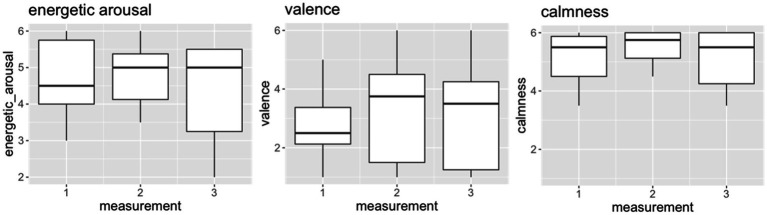
The indicated values (1–6) of the dimensions energetic arousal, valence and calmness for measurement timepoint 1 (PRE), measurement timepoint 2 (POST) and measurement timepoint 3 (FoUp1h) for patient 1. The boxplots display the median and the 2nd and 3rd quartile.

In patient 1 (Figure 1), our analyses showed a difference between the three measurement timepoints for energetic arousal, with the median score increasing from 4.5 (PRE) to 5.0 (POST) l. The valence scores increased from 2.5 (PRE) to 3.75 (POST), and the calmness scores increased from 5.5 (PRE) to 5.75 (POST). This indicates that patient 1 felt more energized, more comfortable, and less restless immediately after ST. The median energetic arousal score was stable at FoUp1h (5.0), while the median scores on valence (3.5) and calmness (5.5) decreased at follow-up.

Small effect sizes were observed for energetic arousal (η^2^ = 0.01) and calmness (η^2^ = 0.02), while no effect was observed for valence (η^2^ = 0.0041).

### Patient 2

**Figure 2:**
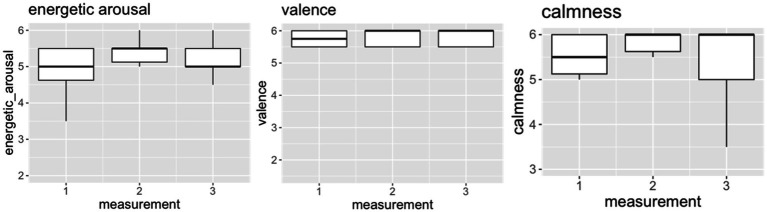
The indicated values (1–6) of the dimensions energetic arousal, valence and calmness for measurement timepoint 1 (PRE), measurement timepoint 2 (POST) and measurement timepoint 3 (FoUp1h) for patient 2. The boxplots display the median and the 2nd and 3rd quartile.

In patient 2 (Figure 2), our analyses showed differences in affective dimensions between the three measurement timepoints, with the median energetic arousal score increasing from 5.0 (PRE) to 5.5 (POST), the median valence score increasing from 5.75 (PRE) to 6.0 (POST), and the median calmness score increasing from 5.5 (PRE) to 6.0 (POST). This indicates that patient 2 felt more energized, more comfortable, and less restless immediately after ST. The scores at FoUp1h were stable for valence (6.0) and in calmness (6.0), while the median energetic arousal score decreased at follow-up (5.0).

Medium effect sizes were observed for energetic arousal (η^2^ = 0.07) and valence (η^2^ = 0.07), but no effect was observed for calmness (η^2^ = 0.000572).

### Patient 10

**Figure 3:**
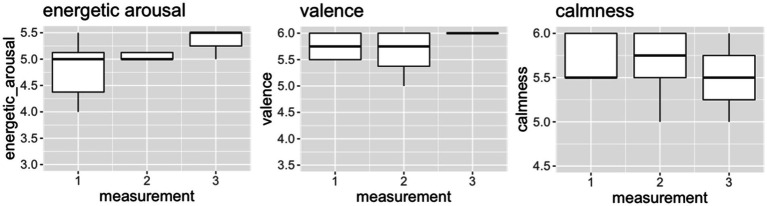
The indicated values (1–6) of the dimensions energetic arousal, valence and calmness for measurement timepoint 1 (PRE), measurement timepoint 2 (POST) and measurement timepoint 3 (FoUp1h) for patient 10. The boxplots display the median and the 2nd and 3rd quartile.

In patient 10 (Figure 3), our analyses showed a difference in calmness between the three measurement timepoints, with the median score increasing from 5.5 (PRE) to 5.75 (POST) and the decreasing to 5.5 again at FoUp1h. For energetic arousal and valence, the median score did not change between the PRE to POST timepoints (5.5 and 5.75, respectively). Furthermore, the median scores for energetic arousal (5.5) and valence (6.0) increased at FoUp1h.

A medium effect size was observed for energetic arousal (η^2^ = 0.06), but no effects were observed for valence (η^2^ = 0.00133) and calmness (η^2^ = 0.00217).

## Discussion

In our study, we evaluated the feasibility of an intensive longitudinal case study design in forensic psychiatry to determine whether forensic patients are content to complete several data assessments per day over the course of several weeks, with feasibility operationalized by the compliance rate. Herein, single-case studies were performed to examine the effects of ST on momentary affective states (energetic arousal, valence, and calmness); the completion of these assessments reflect one aspect of feasibility. The findings reveal effects of ST in forensic psychiatric patients with different conditions.

On the one hand, our results provide insight into the feasibility of an intensive longitudinal design with respect to the compliance rate. Intensive longitudinal case studies, however, also bring new challenges, such as a high effort for the patients and thus a declining compliance rate. The compliance rate of >63% was only based on three patients. Concerning the challenge due to the compliance rate, our findings showed that out of 10 participants, three subjects dropped out before the end of the study. Four additional participants did not provide sufficient data for analysis. Ultimately, three patients provided data that were suitable for analysis. Thus, the compliance rate was ≤37% in seven out of 10 patients. Our data show that single-case studies are able to express heterogeneity more precisely. Regarding the low compliance rate in our study or other studies with special populations such as forensic psychiatric subjects (7), the very different daily routines of the participants made it difficult to determine the appropriate measurement timepoints at which all participants could be reached for the FoUp1h measurement timepoint. Of the three intervention measurement timepoints, the FoUp1h was extremely difficult to implement (see Table 1) since the patients had to be actively sought. Reaching the participants for this intervention measurement timepoint was extremely difficult, while reaching the subjects for the PRE and POST measurement timepoints was easier.

On the other hand, our single-case analyses reveal that the results are heterogeneous. Except for energetic arousal, the effects are heterogeneous overall. The rate of missing data was high in our study. We did not have a sufficient amount of data per patient for a proper single-case analysis. Power analyses revealed that the study was underpowered in all three patients (patient 1: 0.39; patient 2: 0.36; patient 10: 0.26). For a sufficiently powered study (0.80), at least 73 data per analysis would have been needed (34). The many missing data can be explained by the fact that a lack of motivation quickly spread. After 2 weeks of the study, the patients had to be extremely motivated to continue filling out the questionnaires. The fourth and last week of the study had the most missing data, which is why the study was terminated after 4 weeks. Ultimately, only the people who participated at enough measurement timepoints could be included. Additionally, the FoUp1h measurement timepoint was a huge barrier because there were only very few patients left at that time. Our low sample size of 10 participants represents the normal sample size in forensic psychiatric ST. Concerning the effects of ST on affective states, none of the three affect dimensions changed significantly between the three measurement timepoints. There were low to medium differences between the three measurement timepoints on energetic arousal, although these differences were nonsignificant. It must be noted that two of the three patients started with high values, and thus, no large increases could be expected. Our findings are partly in line with previous results: an intervention study showed positive associations between a sport and exercise therapy program (3 months with a 90-min session twice a week) and affective states in a single-case study with one person diagnosed with posttraumatic stress disorder (35). As the duration of the program increased, positive affective states were perceived more often (35). A meta-analysis examining within-person changes in affective responses to physical activity in people with depression shows that acute bouts of physical activity moderately improve affective states (36). The discrepancy between these findings and the findings of our study is due to the fact that forensic psychiatric studies present challenges that differ from the challenges associated with studies among patients with mental illness without a forensic connection. Forensic psychiatry has stricter rules, and thus, there is less freedom in the design and implementation of studies in ST.

Further research in this area is needed. This intervention provides initial approaches with the special population of forensic psychiatric patients.

### Limitations

It should be noted that repeated-measures ANOVA was conducted with normality and variance homogeneity partially being present since other statistical methods would not have produced more significant results either. Moreover, it was used for data that all came from the same individual. There is a possibility that this could have limited the variability between measurements and led to inaccurate results.

In addition, it must be noted that a sufficiently powered study (0.80) was not achievable due to the small amount of data.

The high baseline values on the affective dimensions for two patients might represent ceiling effects.

Forensic psychiatric patients are heterogeneous, and the individual effects of interventions on their affective states are also heterogeneous. Therefore, the results obtained herein can hardly be generalized. Even patients with the same diagnosis can present a different symptoms and medication regimes; therefore, it is extremely challenging to compare or equate one forensic patient to another.

## Implication

This study suggests that an intensive longitudinal case study design in forensic psychiatry is feasible and that ST has small to medium short-term effects on affective states in individual patients who show a compliance rate of >63%. Compared to a between-subject design, we only used data of patients with an appropriate compliance rate for the evaluation.

However, the study design needs intensive revision to increase the compliance rate for particular study samples such as forensic patients. For example, a longer study duration and a survey of PRE and POST measurement timepoints alone could ensure a better compliance rate. Future studies should consider that data collection timepoints must be firmly integrated into the patients’ schedule (especially for the FoUp1h measurement). In collaboration with the facility, an individualized plan should be developed for each patient so that they are able to complete each questionnaire at every measurement timepoint.

Especially for longer-term data collection, participants of the study could be provided with a study smartphone (digital data collection). They could be reminded about the digital questionnaire via a signal tone, thereby enabling data collection even on days without ST to evaluate whether and to what extent patients feel better on days with ST.

Furthermore, participation in a study is only meaningful for patients who regularly participate in ST. In our study, it was shown that patients who regularly participated in ST also completed this study with a compliance rate of >63%. Patients who did not participate regularly in ST were more likely to drop out of the study or not to participate conscientiously. If only patients who regularly participate in ST are included in a study, less missing data can be expected. Data collection on at least 2 days of ST per week (à 3 measurement timepoints) over a period of at least 12 weeks is reasonable and realistic.

Another option is to stagger the reward (a small reward after each week and a large reward at the end of the study).

Further studies, e.g., multicenter studies, need to find out how and to whom the results can be generalized (patients with same diagnosis, same sex, similar age, similar medication, etc.).

## Conclusion

Due to the abovementioned challenges (heterogeneity, low sample size, low and compliance rate) in conducting studies in ST in forensic psychiatry, intensive longitudinal case studies are a possible approach to address heterogeneity and the low sample size. To counteract the high effort of data delivery for the patients and the associated low compliance rate, a change of the study design (e.g., digital data collection) and a fixed establishment of the measurement timepoints in the patients’ schedule could improve the power of intensive longitudinal case studies.

## Data availability statement

The raw data supporting the conclusions of this article will be made available by the authors, without undue reservation.

## Ethics statement

Ethical review and approval was not required for the study on human participants in accordance with the local legislation and institutional requirements. The patients/participants provided their written informed consent to participate in this study.

## Author contributions

VR and MK contributed to the conception and design of the study and wrote sections of the manuscript. VR organized the database, performed the statistical analysis, and wrote the first draft of the manuscript. All authors contributed to the article and approved the submitted version.

## Funding

This work was supported by the Centre for Psychiatry Reichenau for financing the third-party project funding and library of the University of Konstanz for paying the fees for open access.

## Conflict of interest

The authors declare that the research was conducted in the absence of any commercial or financial relationships that could be construed as a potential conflict of interest.

## Publisher’s note

All claims expressed in this article are solely those of the authors and do not necessarily represent those of their affiliated organizations, or those of the publisher, the editors and the reviewers. Any product that may be evaluated in this article, or claim that may be made by its manufacturer, is not guaranteed or endorsed by the publisher.
