# Supplementary material for: Does sports therapy affect momentary affective states? Feasibility of intensive longitudinal case studies in forensic psychiatry
Source: Front Psychiatry. 2023 May 12;14:1111602. doi: 10.3389/fpsyt.2023.1111602 (PMC10217360; doi:10.3389/fpsyt.2023.1111602)
Supplement: Supplementary file 1 [file Data_Sheet_1.docx]

1. **One-time initial questionnaire:**

| **Im Allgemeinen fühle ich mich…** | stimme ganz und gar nicht zu | stimme nicht zu | stimme eher nicht zu | stimme eher zu | stimme zu | stimme voll und ganz zu |
| --- | --- | --- | --- | --- | --- | --- |
| …zufrieden. | ◻ | ◻ | ◻ | ◻ | ◻ | ◻ |
| …ausgeruht. | ◻ | ◻ | ◻ | ◻ | ◻ | ◻ |
| …ruhelos. | ◻ | ◻ | ◻ | ◻ | ◻ | ◻ |
| …schlecht. | ◻ | ◻ | ◻ | ◻ | ◻ | ◻ |
| …schlapp. | ◻ | ◻ | ◻ | ◻ | ◻ | ◻ |
| …gelassen. | ◻ | ◻ | ◻ | ◻ | ◻ | ◻ |
| …müde. | ◻ | ◻ | ◻ | ◻ | ◻ | ◻ |
| …gut. | ◻ | ◻ | ◻ | ◻ | ◻ | ◻ |
| …unruhig. | ◻ | ◻ | ◻ | ◻ | ◻ | ◻ |
| …munter. | ◻ | ◻ | ◻ | ◻ | ◻ | ◻ |
| …unwohl. | ◻ | ◻ | ◻ | ◻ | ◻ | ◻ |
| …entspannt. | ◻ | ◻ | ◻ | ◻ | ◻ | ◻ |

|  | stimme ganz und gar nicht zu | stimme nicht zu | stimme eher nicht zu | stimme eher zu | stimme zu | stimme voll und ganz zu |
| --- | --- | --- | --- | --- | --- | --- |
| Ich stehe häufig vor Unerledigtem und weiß nicht, was ich tun soll. | ◻ | ◻ | ◻ | ◻ | ◻ | ◻ |
| Es fällt mir schwer, mich selbst zu unterhalten. | ◻ | ◻ | ◻ | ◻ | ◻ | ◻ |
| Viele Dinge, die ich tun muss, wiederholen sich und sind monoton. | ◻ | ◻ | ◻ | ◻ | ◻ | ◻ |
| Es braucht mehr Anregung, um mich in die Gänge zu bringen, als bei den meisten anderen Menschen. | ◻ | ◻ | ◻ | ◻ | ◻ | ◻ |
| Die meisten Dinge, die ich mache, motivieren mich nicht. | ◻ | ◻ | ◻ | ◻ | ◻ | ◻ |
| In den meisten Situationen fällt es mir schwer, etwas zu finden, was ich tun oder sehen kann, um mein Interesse aufrechtzuerhalten. | ◻ | ◻ | ◻ | ◻ | ◻ | ◻ |
| Einen Großteil der Zeit sitze ich einfach rum und mache nichts. | ◻ | ◻ | ◻ | ◻ | ◻ | ◻ |
| Ich fühle mich halb tot und stumpf, es sei denn, ich mache etwas Aufregendes oder sogar Gefährliches. | ◻ | ◻ | ◻ | ◻ | ◻ | ◻ |

1. **Recurring questionnaire: Current state and boredom.**

**In diesem Moment fühle ich mich…**

| sehr  müde | ◻ | ◻ | ◻ | ◻ | ◻ | ◻ | sehr  wach |
| --- | --- | --- | --- | --- | --- | --- | --- |
|  |  |  |  |  |  |  |  |
| sehr  zufrieden | ◻ | ◻ | ◻ | ◻ | ◻ | ◻ | sehr unzufrieden |
|  |  |  |  |  |  |  |  |
| sehr  unruhig | ◻ | ◻ | ◻ | ◻ | ◻ | ◻ | sehr  ruhig |
|  |  |  |  |  |  |  |  |
| sehr  energiegeladen | ◻ | ◻ | ◻ | ◻ | ◻ | ◻ | sehr  energielos |
|  |  |  |  |  |  |  |  |
| sehr  unwohl | ◻ | ◻ | ◻ | ◻ | ◻ | ◻ | sehr  wohl |
|  |  |  |  |  |  |  |  |
| sehr  entspannt | ◻ | ◻ | ◻ | ◻ | ◻ | ◻ | sehr  angespannt |

**Im Moment bin ich…**

| sehr  gelangweilt | ◻ | ◻ | ◻ | ◻ | ◻ | ◻ | ◻ | ◻ | ◻ | ◻ | überhaupt nicht gelangweilt |
| --- | --- | --- | --- | --- | --- | --- | --- | --- | --- | --- | --- |
